# Combined contact force and local impedance dynamics during repeat atrial fibrillation catheter ablation

**DOI:** 10.3389/fphys.2022.1001719

**Published:** 2022-10-13

**Authors:** Fares-Alexander Alken, Katharina Scherschel, Ann-Kathrin Kahle, Mustafa Masjedi, Christian Meyer

**Affiliations:** ^1^ Division of Cardiology, Angiology and Intensive Care, cNEP, Cardiac Neuro- and Electrophysiology Research Group, EVK Düsseldorf, Düsseldorf, Germany; ^2^ Department of Neurophysiology, Heinrich-Heine-University Düsseldorf, Medical Faculty, cNEP, Cardiac Neuro- and Electrophysiology Research Consortium, Düsseldorf, Germany; ^3^ Department of Cardiology, Pulmonology and Vascular Medicine, Medical Faculty, University Hospital Düsseldorf, Düsseldorf, Germany

**Keywords:** atrial fibrillation, contact force, local impedance, atrial arrhythmia, catheter ablation, myocardial scar

## Abstract

**Background:** Optimal lesion formation during catheter-based radiofrequency current (RFC) ablation depends on electro-mechanical tip-tissue coupling measurable *via* contact force (CF) and local impedance (LI) monitoring. We aimed to investigate CF and LI dynamics in patients with previous atrial fibrillation (AF) ablation who frequently present with heterogenous arrhythmia substrate.

**Methods:** Data from consecutive patients presenting for repeat AF or atrial tachycardia ablation using a novel open-irrigated single-tip ablation catheter were studied. RFC applications were investigated regarding CF, LI and the maximum LI drop (∆LI) for evaluation of ablation efficacy. ∆LI > 20 Ω was defined as a successful RFC application.

**Results:** A total of 730 RFC applications in 20 patients were analyzed. Baseline CF was not associated with baseline LI (R = 0.06, *p* = 0.17). A mean CF < 8 g during ablation resulted in lower ∆LI (<8 g: 13 Ω vs. ≥ 8 g: 16 Ω, *p* < 0.001). Baseline LI showed a better correlation with ∆LI (R = 0.35, *p* < 0.001) compared to mean CF (R = 0.17, *p* < 0.001). Mean CF correlated better with ∆LI in regions of low (R = 0.31, *p* < 0.001) compared to high (R = 0.21, *p* = 0.02) and intermediate voltage (R = 0.17, *p* = 0.004). Combined CF and baseline LI predicted ∆LI > 20 Ω (area under the receiver operating characteristic curve (AUC) 0.75) better compared to baseline LI (AUC 0.72), mean CF (AUC 0.60), force-time integral (AUC 0.59) and local bipolar voltage (0.55).

**Conclusion:** Combination of CF and LI may aid monitoring real-time catheter-tissue electro-mechanical coupling and lesion formation within heterogenous atrial arrhythmia substrate in patients with repeat AF or atrial tachycardia ablation.

## Introduction

Optimal lesion formation during catheter-based radiofrequency current (RFC) ablation has been known to depend on sufficient mechanical contact and electrical coupling for more than two decades (Ariyarathna et al., 2018). Measurement of beat-to-beat tip-tissue contact force (CF) is widely implemented today as a surrogate parameter for catheter stability (Calkins et al., 2018). However, as a physical parameter CF does not give insights into tissue response during ablation.

The applied electrical current inducing tissue heating is dependent on the resistive load during catheter tip-tissue contact, with measurement attempted *via* impedance by different technologies: classical transthoracic impedance measurement by RFC generators (between catheter tip and indifferent surface electrodes) is limited by significant inter-patient variability regarding torso impedance as well as intra-patient variability subject to the respiratory phase (Borganelli et al., 1992; Barkagan et al., 2018; Chu et al., 2022). Measurement of local impedance (LI) is conducted by driving a non-stimulatory current between a distal and proximal electrode. It has gained interest as it resembles the resistive load at the catheter tip which is proportional to the tissue-catheter contact (Barkagan et al., 2018; Sulkin et al., 2018). Therefore, when compared to generator impedance, baseline LI shows greater variation between blood pool and tissue (Sulkin et al., 2018; Gunawardene et al., 2019). A strong relationship between the LI drop during ablation (∆LI) and efficacy of lesion formation has been demonstrated (Gunawardene et al., 2019; Garrott et al., 2020; Das et al., 2021; Solimene et al., 2021), with a ∆LI > 20 Ω being proposed to result in sufficient lesion formation according to previous clinical and experimental studies (Garrott et al., 2020; Szegedi et al., 2021; Ikenouchi et al., 2022; Sasaki et al., 2022).

Low voltage zones indicate fibrofatty infiltration and contribute to initiation and maintenance of atrial fibrillation (AF) and atrial tachycardia (AT). This is especially of interest in repeat ablation procedures, where reconnected pulmonary veins as well as reconduction of previously ablated or new low voltage zones are frequently encountered due to myocardial scarring (De Pooter et al., 2019; Huo et al., 2020). Creating durable ablation lesions in these low voltage zones remains of importance for ablation success but is challenged by several factors: Scar tissue offers heterogenous thermodynamic properties affecting resistive and conductive heating. Lower resistivity of collagen results in reduced heating as well as shunting of electrical current to blood, while adipose tissue is often present in scar areas providing higher electrical resistivity (Barkagan et al., 2019; Tao et al., 2019). We therefore aimed to investigate how CF correlates with changes in LI during repeat AF or AT ablation within heterogenous arrhythmia substrate.

## Methods

### Clinical cohort

In this explorative single-center series, consecutive patients presenting for catheter ablation due to continuous symptomatic atrial arrhythmia (AF or AT) after previous AF ablation were analyzed retrospectively. This analysis was approved by the local ethics committee (Ärztekammer Nordrhein, 201/2022) and written informed consent was obtained from all patients.

### Electrophysiological study setup and catheter ablation procedure

The procedures were carried out in accordance to existing consensus statements as well as previously published protocols (Calkins et al., 2018; Alken et al., 2019). In short, patients underwent the procedure in a fasting state under conscious sedation using propofol or midazolam and fentanyl. Hemodynamic monitoring consisted of continuous assessment of saturation, non-invasive blood pressure as well as surface and intracardiac electrocardiogram recordings. A single transseptal puncture was conducted with fluoroscopic guidance and catheters were introduced into the left atrium using a fixed curve long sheath (SL0, 8-F; St. Jude Medical, for ablation catheter) and a long steerable sheath (Zurpaz™, medium curl, 8.5-F, Boston Scientific, for mini basket catheter). The catheter setting consisted of a steerable 6F decapolar catheter (Inquiry™, 5 mm spacing; St. Jude Medical, Saint Paul, MN, United States) positioned in the coronary sinus and serving as the reference of the Rhythmia™ 3-D electroanatomical mapping system (Boston Scientific Corporation, Marlborough, MA, United States) as well as an expandable, open-irrigated 64-pole mini basket mapping catheter (IntellaMap Orion™, Boston Scientific) comprising 8 splines with 8 electrodes for creation of ultra-high density maps. Furthermore, a novel open-irrigated single-tip ablation catheter (IntellaNav StablePoint™, Boston Scientific, Marlborough, MA, United States) capable of continuously assessing CF (translation of force to inductive sensors *via* a spring) and LI (non-stimulatory alternating current between tip and proximal ring electrode) was introduced (Garrott et al., 2020). Activated clotting time was monitored after first access of the left atrium, targeting a level >300 s.

A blood pool reference value was established without tissue contact for both CF and LI before ablation onset. In general, persistence of pulmonary vein isolation as well as previously set linear lesions was checked and discovered gaps were ablated. RFC applications were delivered with a standard power of 30 W, while adjusting power to 25 W at the posterior left atrial wall and 40 W at the cavotricuspid isthmus where applicable. The irrigation rate was maintained at 17–30 ml/min for up to 120 s and the upper temperature limit was set at 48°C. Additional substrate modification *via* ablation of complex fractionated atrial electrograms was conducted at the operator’s discretion. In macro-reentrant AT the critical isthmus was detected and ablated consecutively. In case of localized or focal AT, the site of earliest activation was targeted. Pacing maneuvers and pharmacological provocation were performed repeatedly to test for arrhythmia non-inducibility. A bidirectional block was confirmed whenever linear lesions were generated. Acute ablation success was defined as non-inducibility of atrial arrhythmias at the end of the procedure.

### Postprocedural radiofrequency current application analysis

To study the biophysics of lesion formation, point-by-point RFC applications were analyzed regarding applied CF, force-time integral and detected LI before onset and during ablation. An automatized export software integrated into the Rhythmia™ system was used to export CF and LI data, while full-length procedure recordings and generated voltage maps were reviewed complementing analysis. Baseline, minimum, maximum and mean CF/LI values as well as the absolute and relative difference between baseline and minimum LI during ablation (maximum absolute LI drop, ∆LI; maximum LI drop in percent, ∆LI%) were measured. To account for inter-patient variability of blood pool LI, the difference between baseline tissue LI and blood pool LI (∆baseline LI) was measured in all patients. Sufficient lesion formation was defined as a ∆LI > 20 Ω according to previous preclinical findings (Garrott et al., 2020; Szegedi et al., 2021; Ikenouchi et al., 2022; Sasaki et al., 2022). CF and LI dynamics were further analyzed considering local bipolar voltage for each RFC application, differentiating between high (>0.5 mV), intermediate (0.1–0.5 mV) and low (<0.1 mV) voltage (Frontera et al., 2018). RFC applications with a duration <10 s, poor signal quality, instable catheter contact or dragged ablation points were excluded from analysis for parameter standardization.

### Statistical analysis

Continuous variables are expressed as mean ± standard deviation or absolute numbers and percentages. In case of non-parametric data, results are provided as medians with interquartile ranges. All analyses were performed using Graphpad Prism 9 (Graphpad Inc., La Jolla, CA, United States), MedCalc (MedCalc Software Ltd., Ostend, Belgium) and Microsoft Excel. For determination of normal distribution, the Shapiro-Wilk test was conducted. In case of paired datasets, a paired t-test was conducted for normally distributed and a Wilcoxon signed-rank test for non-normally distributed datasets. For comparison of unpaired datasets, an unpaired t-test was conducted in case of normal and the Mann-Whitney U test in case of non-normal distribution. A Kruskal–Wallis test was applied for unpaired comparison of more than two groups in case of non-normal distribution and an one-way analysis of variance (ANOVA) in case of normal distribution of datasets.

Correlation analysis was conducted to investigate the relationship of baseline LI to ∆LI and mean CF using Pearson correlation coefficients for normally distributed and Spearman correlation for non-normally distributed datasets. A Pearson R or Spearman R coefficient was calculated, where a positive value indicates a positive and a negative value a negative linear relation. Linear regression analysis was conducted to determine a fitted regression graph with 95% confidence bands. Simple and multiple logistic regression analysis was performed for prediction of a ∆LI > 20 Ω which has been described to result in sufficient lesion formation (Garrott et al., 2020). A *p*-value <0.05 was considered statistically significant.

## Results

### Baseline contact force and local impedance characteristics

Baseline patient as well as procedural characteristics are displayed in Table 1, 2. Out of 1488 RFC applications in 20 consecutive patients, 730 (49%) were eligible for analysis, of which 93 % were located in the left atrium. Ablation was conducted at the pulmonary veins (54%), left atrial anterior wall (15%), left atrial roof (9%), mitral isthmus (6%), interatrial septum (5%), left atrial posterior wall (3%), left atrial appendage (1%), cavotricuspid isthmus (6%), coronary sinus and superior vena cava (1%). Acute procedural success was achieved in all patients and no catheter-related periprocedural complications were observed.

**TABLE 1:** Baseline patient parameters.

**Variable**	**Total (*n* = 20)**
Age [years]	66±11
Female sex, n (%)	8 (40)
Time since atrial fibrillation diagnosis [years]	8±6
Paroxysmal atrial fibrillation, n (%)	9 (45)
Persistent atrial fibrillation, n (%)	11 (55)
Atrial tachycardia, n (%)	10 (50)
Prior ablation procedures [n]	1.7±0.9
CHA_2_DS_2_-VASc-Score [n]	3 (2-4)
Arterial hypertension, n (%)	18 (90)
Diabetes mellitus, n (%)	2 (10)
Hyperlipidemia, n (%)	6 (30)
Prior stroke / transient ischemic attack, n (%)	1 (5)
Coronary artery disease, n (%)	7 (35)
Heart failure, n (%)	8 (40)
Left ventricular ejection fraction [%]	49 (43-60)
Ischemic cardiomyopathy, n (%)	2 (10)
Dilated cardiomyopathy, n (%)	1 (5)
Hypertrophic cardiomyopathy, n (%)	1 (5)
Tachymyopathy, n (%)	4 (20)
Prior revascularization, n (%)	4 (20)

Variables are expressed as absolute values with percentages, mean with standard deviation or median with interquartile ranges.

**TABLE 2:** Procedure characteristics.

**Variable**	**Total (*n* = 20)**
Procedure time [min]	225 (128-270)
Fluoroscopy time [min]	17 (7-26)
Dose area product [cGy*cm^2^]	75 (47-117)
Number of radiofrequency deliveries analyzed per patient [n]	35 (22-46)
Total ablation time per patient [min]	20 (11-34)
Ablation duration per radiofrequency delivery [s]	20 (14-29)
Repeat pulmonary vein isolation, n (%)	15 (75)
Left atrial tachycardia ablation, n (%)	11 (55)
Right atrial tachycardia ablation, n (%)	4 (20)

Variables are expressed as absolute values with percentages or median with interquartile ranges.

Baseline blood pool LI serving as a non-contact reference was similar compared to left and right atrial baseline LI (blood pool: median 146 (interquartile range 138-156) Ω vs left atrial: 147 (136-163) Ω and right atrial: 146 (132-165) Ω, Kruskal-Wallis test, p = 0.95). Median baseline LI was 146 (132-165) Ω and applied baseline CF was 9 (5-15) Ω. Baseline CF and LI did not show a significant correlation in the overall cohort (Spearman correlation, R = 0.06, *p* = 0.17) as well as when distinguishing between high (Spearman correlation, R = 0.17, *p* = 0.23), intermediate (Spearman correlation, R = −0.18, *p* = 0.054) and low voltage zones (Spearman correlation, R = 0.19, *p* = 0.23). ∆Baseline LI was not associated with baseline CF (Spearman correlation, R = 0.04, *p* = 0.39), while 45% of analyzed RFC applications displayed lower baseline LI compared to blood pool LI.

### Impact of contact force and baseline local impedance on the local impedance drop

**FIGURE 1:**
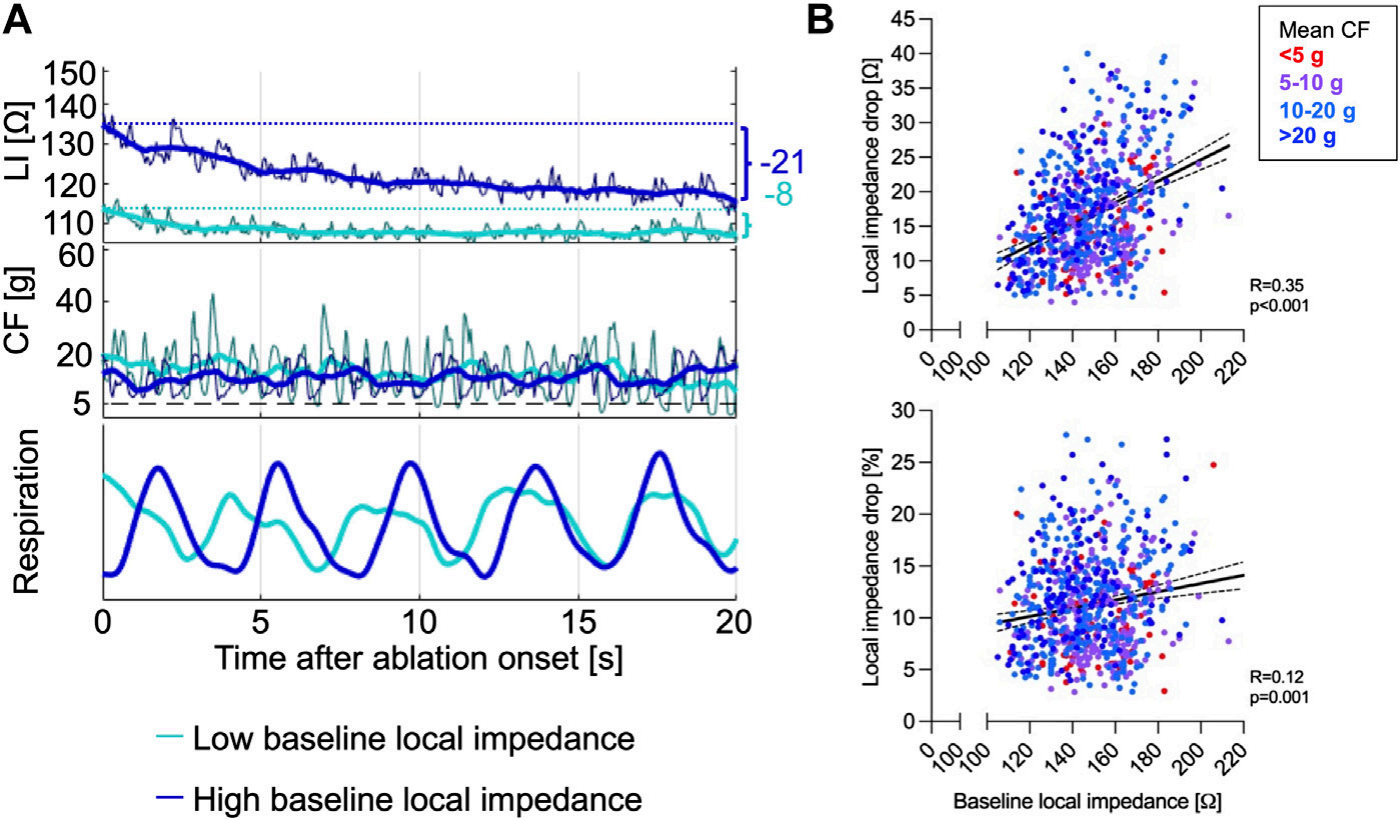
**(A)** Representative CF and LI raw as well as time-smoothed averaged recordings in a single patient during ablation are displayed. High baseline LI (dark blue) predicted increased ∆LI, while lower baseline LI (light blue) resulted in low δLI despite similar energy output of 30 W and adequate CF levels ≥8 g. **(B)** Baseline LI correlated better with ∆LI (upper panel) compared to the ∆LI% (lower panel). CF, contact force; LI, local impedance; ∆LI, absolute local impedance drop; ∆LI%, relative local impedance drop.

The median ∆LI per RFC application displayed at 15 (interquartile range 11–21) Ω and ∆LI% at 11 (8–14) %. High baseline LI predicted an increased δLI and correlated better with ∆LI (Spearman correlation, R = 0.35, *p* < 0.001) compared to ∆LI% (Spearman correlation, R = 0.12, *p* = 0.001, Figures 1A,B). ∆LI increased by 1 Ω with every 6.5 Ω baseline LI gain.

**FIGURE 2:**
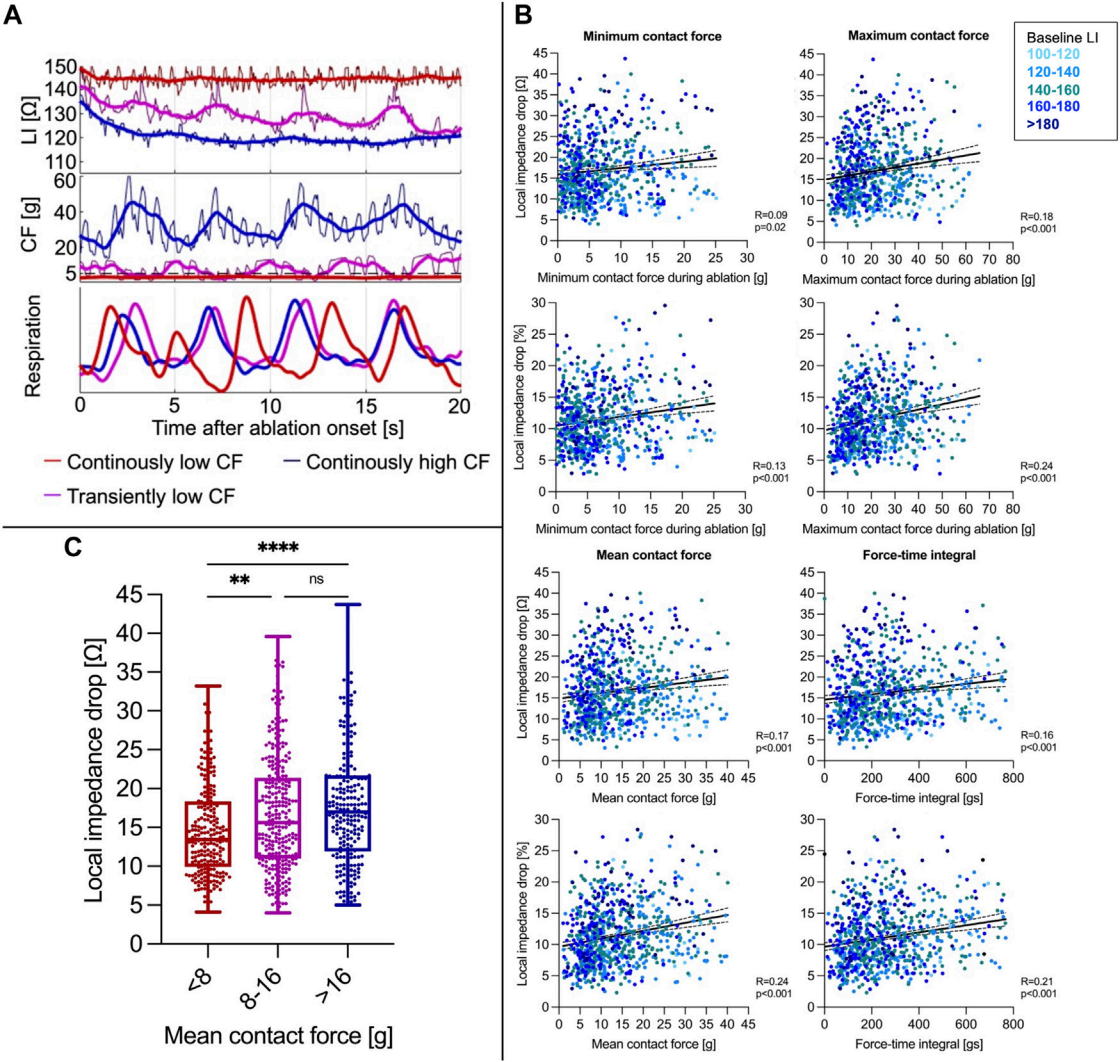
**(A)** Representative CF and LI raw as well as time-smoothed averaged recordings in a single patient during ablation are displayed. In regions of similar baseline LI, high constant CF (blue curve) resulted in a rapid δLI after approximately 4 s, whereas intermittent decreases of CF to 0 g due to inspiration (violet) went along with LI increases reflecting transient catheter-tissue contact loss with overall similar δLI. Continuously low CF (red) resulted in a reduced ∆LI. **(B)** Correlation of minimum/maximum/mean CF during ablation and force-time integral with ∆LI (upper panel) and ∆LI% (lower panel). **(C)** A mean CF < 8 g was concomitant with a reduced LI drop, whereas increases beyond 8 g did not further increase the LI drop. **p ≤ 0.01; ****p ≤ 0.0001. For abbreviations see Figure 1.

The median value of mean CF per RFC application was 11 (7–19) g. Transient catheter-tissue contact loss due to respiratory motion was reflected by low CF and simultaneous LI increases ultimately resulting in reduced ∆LI (Figure 2A). Mean CF and force-time integral showed a weaker correlation with ∆LI (Spearman correlation, CF: R = 0.17, *p* < 0.001; force-time integral: R = 0.16, *p* < 0.001) and with ∆LI% (Spearman correlation, CF: R = 0.24, *p* < 0.001; force-time integral: R = 0.21, *p* < 0.001, Figure 2B) compared to baseline LI.

For determination of the optimal mean CF cut-off value to achieve a ∆LI > 20 Ω, receiver operating characteristic analysis was conducted and revealed a mean CF level ≥8 g as the best predictor with overall limited sensitivity (0.75) and specificity (0.39). RFC applications with a mean CF < 8 g displayed lower ∆LI (< 8 g: 13 (9-18) Ω vs. <8 g: 16 (11-22) Ω, Mann-Whitney test, *p* < 0.001). However, mean CF rises beyond 8 g did not further alter ∆LI (8-16 g: 16 (11-21) Ω vs. > 16 g: 17 (12-22) Ω, Mann-Whitney test, *p* = 0.24, Figure 2C). A force-time integral <400 gs was also associated with lower ∆LI (<400 gs: 14 (10-20) Ω vs. <400 gs: 18 (12-24) Ω, Mann-Whitney test, *p* < 0.00).

### Influence of underlying myocardial substrate on force-impedance dynamics

**FIGURE 3:**
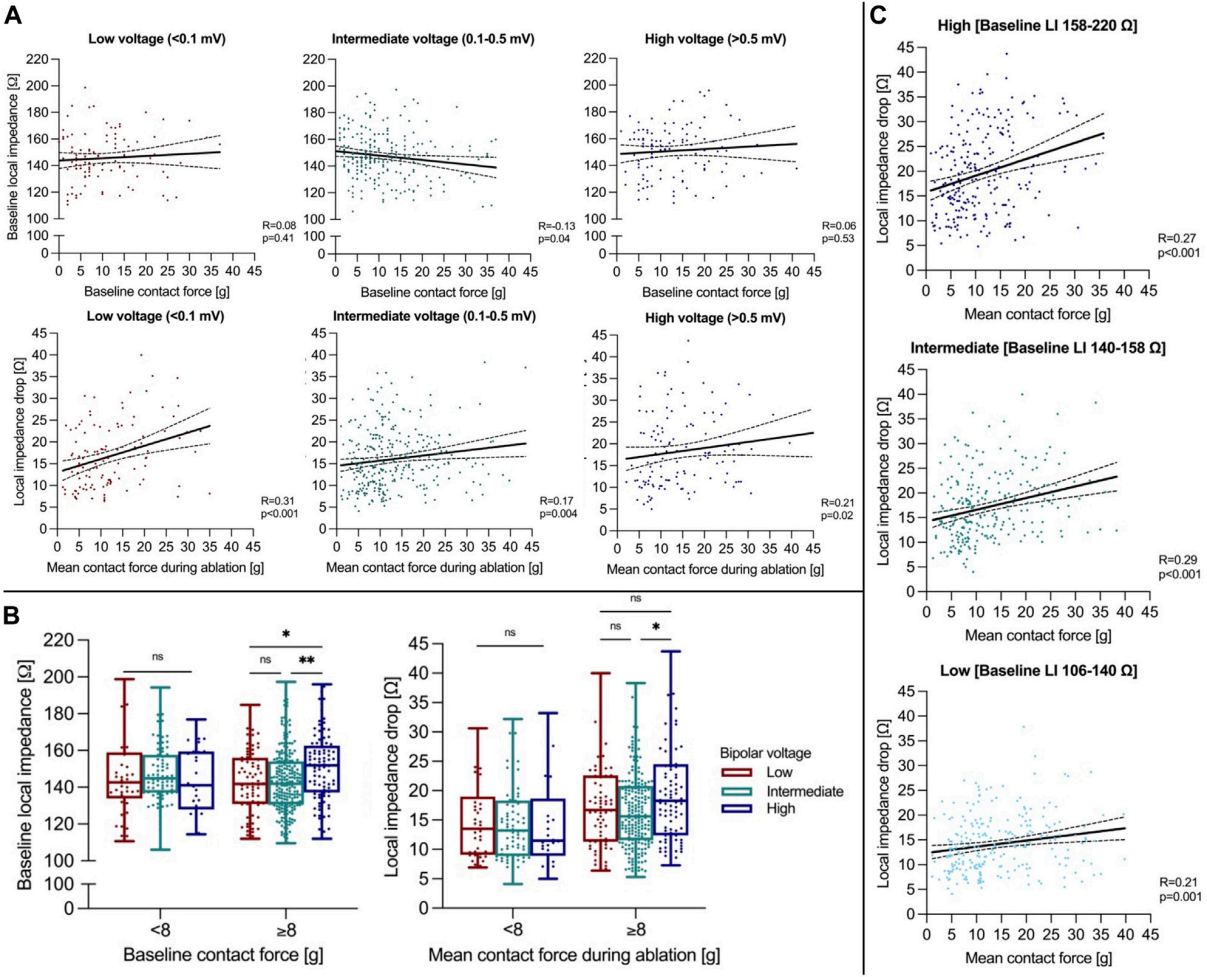
**(A)** Correlation analysis of baseline CF to LI (upper panel) and mean CF to the ∆LI (lower panel) is shown. **(B)** Baseline LI and ∆LI during ablation are displayed depending on local bipolar voltage and applied CF classified by the estimated mean CF cut-off of 8 g. **(C)** Correlation analysis of mean CF to ∆LI depending on underlying baseline LI terciles. *p ≤ 0.05; ***p ≤ 0.001. For abbreviations see Figure 1.

A subset of 547 RFC applications was analyzed regarding underlying local bipolar voltage. Baseline LI was higher in regions of high vs intermediate or low voltage (high: 152 (136-163) Ω vs. intermediate: 143 (132-155) Ω vs. low: 142 (131-156) Ω, Kruskal-Wallis test, p*p* < 0.0001), with applied baseline CF being higher in the high compared to the low voltage group (high: 12 (7-19) g vs. intermediate: 9 (6-16) g vs low: 8 (4-15) g, Kruskal-Wallis test, *p* = 0.02). Baseline CF did not correlate to baseline LI in high/low voltage regions and displayed a weak negative correlation in intermediate voltage regions (Spearman correlation, high: R = 0.06, *p* = 0.53; intermediate: R = −0.13, *p* = 0.04; low: R = 0.08, *p* = 0.41, Figure 3A).

Overall median ∆LI was higher in regions of high compared to intermediate voltage and was similar compared low voltage regions (high: 17 (11-24) Ω vs. intermediate: 15 (11-20) Ω vs. low: 16 (10-22) Ω, Kruskal-Wallis test, *p* = 0.02). Applied mean CF was greater in regions of high compared to low voltage (high: 13 (8-21) g vs. intermediate: 11 (7-18) vs. low: 10 (6-16) g, Kruskal-Wallis test, *p* = 0.007). Mean CF correlated better with ∆LI in regions of low compared to high and intermediate voltage (Spearman correlation, high: R = 0.21, *p* = 0.02; intermediate: R = 0.17, *p* = 0.004; low: R = 0.31, *p* < 0.001). When applying a mean CF cut-off level of 8 g, ∆LI was higher in regions of high compared with intermediate voltage with CF levels ≥8 g (high: 18 (12-25) Ω vs. intermediate: 16 (12-21) Ω vs. low: 17 (11-23) Ω, Kruskal-Wallis test, *p* = 0.04). However, no differences were observed for a mean CF < 8 g between regions (high: 12 (9-19) Ω vs. intermediate: 13 (9-18) Ω vs. low: 14 (9-19) Ω, Kruskal-Wallis test, *p* = 0.72, Figure 3B). When analyzing for underlying baseline LI, mean CF correlated better to ∆LI in the high (158 ‐ 220 Ω) and intermediate baseline LI tercile (140-158 Ω) compared to the low tercile (106-140 Ω, Figure 3C). While mean CF was higher in the low compared to the intermediate and high baseline LI tercile (high: 10 (6-16) g vs. intermediate: 10 (7-17) g vs. low: 11 (8-20) g, Kruskal-Wallis test, *p* < 0.001), ∆LI was elevated the most in the high baseline LI tercile (high: 18 (13-25) Ω vs. intermediate: 16 (12-22) Ω vs. low: 14 (10-18) Ω, Kruskal-Wallis test, *p* < 0.001).

### Prediction of a local impedance drop >20 Ω

**FIGURE 4:**
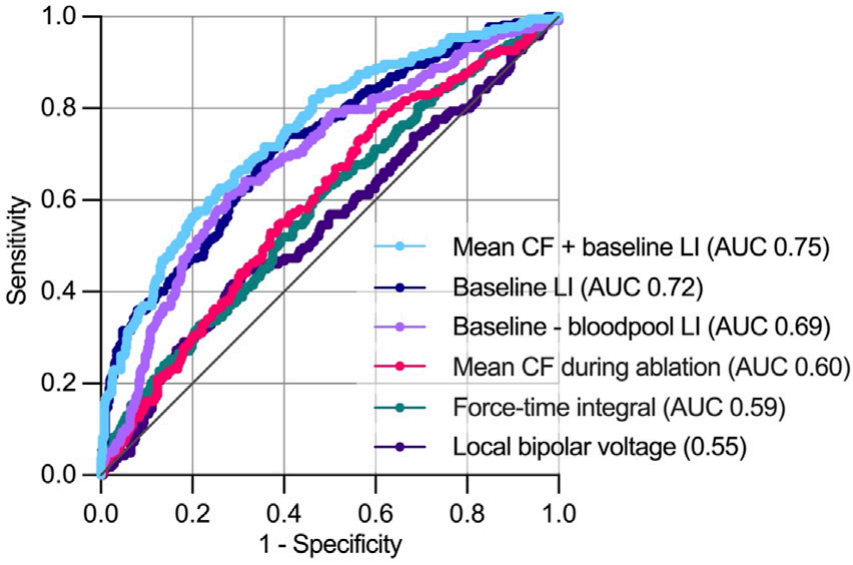
Predictors of a local impedance drop >20 Ω during ablation. During receiver operating characteristics analysis, combined mean CF and baseline LI showed the highest area under the receiver operating characteristics curve for predicting a >20 Ω. AUC, area under curve. For further abbreviations see Figure 1.

A ∆LI > 20 Ω was reached in 29.8% of RFC applications. Receiver operating characteristic analysis displayed combined CF and baseline LI as the best predictor for ∆LI > 20 Ω (area under the receiver operating characteristic curve (AUC) 0.75 [confidence interval (CI) 0.71–0.79], negative/positive predictive value 0.76/0.66, *p* < 0.001) compared to baseline LI [AUC 0.72 (CI 0.68–0.76), negative/positive predictive value 0.84/0.44, *p* < 0.001], ∆baseline LI [AUC 0.69 (CI 0.65–0.73), negative/positive predictive value 0.80/0.50, *p* < 0.001], mean CF (AUC 0.60 (0.55–0.64), negative/positive predictive value 0.79/0.35, *p* < 0.001), force-time integral [AUC 0.59 (CI 0.54–0.63), negative/positive predictive value 0.75/0.35], *p* = 0.001) and bipolar voltage [AUC 0.55 (CI 0.49–0.60), negative/positive predictive value 0.64/0.53, *p* = 0.01, Figure 4].

## Discussion

In this single-center retrospective explorative study, we present for the first time an initial experience of combined CF and LI dynamics in patients undergoing repeat AF or AT ablation. The major findings consist of the following: 1) Applied baseline CF is not significantly associated with baseline LI levels. 2) A minimum mean CF level of 8 g increases ∆LI resembling stable contact, while further CF increases beyond 8 g do not additionally affect ∆LI. 3) Combined mean CF during ablation and baseline LI predicts ∆LI as a surrogate for lesion formation, with the highest ∆LI being observed in regions of high voltage and high baseline LI.

### Biophysical rationale of combined force and impedance assessment

The relevance of sufficient tip-tissue contact for effective lesion formation has been described more than two decades ago (Organ, 1976; Haines, 1991, 2004). CF provides physical feedback on mechanical tip-tissue contact and cardiac as well as respiratory motion (Ariyarathna et al., 2018). Multidimensional indices incorporating parameters additional to CF were introduced aiming for real-time lesion formation estimation during ablation, while lacking information on tissue response (Hussein et al., 2018; Taghji et al., 2018). Here, LI measurement can complement CF: According to Ohm’s law, transmitted current density relies on the electrical field and local tissue resistivity (Garrott et al., 2020; Chu et al., 2022). As power output is fixed by many ablation generators, baseline LI reflects resistive heating potential (Shapira-Daniels et al., 2019) and the degree of ∆LI mirrors volumetric tissue heating during ablation (Barkagan et al., 2018; Sulkin et al., 2018; Garrott et al., 2020). Therefore, combining CF and LI may improve real-time evaluation of lesion formation as electro-mechanical tip-tissue coupling can be assessed simultaneously. This may also contribute to improved safety, as avoiding high mechanical force as well as myocardial tissue heating *via* CF and ∆LI assessment may reduce the risk of steam pops or cardiac perforation (Ariyarathna et al., 2018; Gunawardene et al., 2019; Garrott et al., 2020). In our analysis, a minimum level of 8 g mean CF during ablation ensured sufficient mechanical tip-tissue catheter contact and resulted in increased ∆LI. This is in accordance with previous experimental and clinical studies on other catheter systems measuring CF only, which proposed cut-off values of 5–20 g (Ariyarathna et al., 2018). Excessive CF application may even lower lesion dimensions as it can impair catheter irrigation and result in excessive catheter-tip heating and charring marked by an impedance increase (Ring et al., 1989; Makimoto et al., 2018). High baseline LI guaranteed electrical coupling and may aid catheter positioning additional to CF in challenging regions such as the coronary sinus (Sulkin et al., 2018).

Baseline LI showed the highest correlation to ∆LI which is in line with recently published experimental (Garrott et al., 2020; Gutbrod et al., 2022) and clinical studies (Martin et al., 2018; Gunawardene et al., 2019; Szegedi et al., 2021; Matsuura et al., 2022; Solimene et al., 2022). Furthermore, according to our results combined mean CF and baseline LI was the best predictor for a ∆LI > 20 Ω in comparison to applied mean CF or baseline LI alone. Therefore, maintaining a mean CF level ≥8 g may be valuable in ensuring sufficient tip-tissue contact, while LI provides information on the degree of electrical coupling and real-time tissue response to RFC applications.

### Value of combined force and impedance assessment during repeat atrial arrhythmia ablation

Several experimental and clinical studies have recently been published reporting experiences of combined CF and LI assessment during index AF ablation (Szegedi et al., 2021; Gutbrod et al., 2022; Solimene et al., 2022; Yasumoto et al., 2022). Single-shot devices have seen increased usage for index AF ablations in recent years (Mulder et al., 2021). Therefore, the strength of combining CF and LI monitoring may lie in repeat AF and AT procedures: Here, previously conducted pulmonary vein isolation may show reconnection in up to 70% of cases (Hindricks et al., 2020). Adjunctive ablation strategies additional to pulmonary vein isolation including linear lesions, posterior wall isolation, ablation of non-pulmonary vein triggers and complex fractionated atrial electrograms may be required (Calkins et al., 2018). Reconduction over previous linear or substrate ablation and development of new low voltage areas are frequent findings during repeat AF ablation, while persistent conduction blocks have been reported to be present in only 21% using conventional ablation setups (Huo et al., 2020).

Therefore, index and repeat ablation procedures as well as ablation within healthy compared to impaired myocardium need to be distinguished when evaluating LI and CF dynamics: In our analysis, baseline LI was lower compared to previous results on index AF procedures, but LI correlated better with ∆LI compared to CF which is in line with published data (Szegedi et al., 2021; Ikenouchi et al., 2022; Solimene et al., 2022; Yasumoto et al., 2022). Furthermore, baseline LI was lower compared to blood pool LI in 45% of RFC applications, whereas an elevation of tissue baseline LI beyond blood pool LI was observed in index AF procedures (Solimene et al., 2022). Median ∆LI was lower at 15 Ω compared to index AF procedures reporting ∆LI ranges between 19–27 Ω. These observations may be explained by two central aspects: First, in our cohort with a mean of 2 prior ablation procedures per patient, 60% of RFC applications were located in areas of low voltage where baseline LI was reduced according to our current and previous results (Gunawardene et al., 2019). Second, lesion formation within scarred myocardial tissue results in irregular and lowered tissue injury due to altered thermodynamic characteristics compared to healthy myocardium (Barkagan et al., 2019; Tao et al., 2019). The target ∆LI of >20 Ω chosen in our analysis was only reached in 30% of RFC applications. Currently proposed target ∆LI values are based on experimental and clinical index AF/atrial flutter procedures aiming for healthy myocardium in most cases. Target ∆LI also differs depending on the ablation location within the left atrium (Chu et al., 2022). Therefore, currently proposed target ∆LI levels using this catheter system may not be appropriate in repeat ablation cohorts but rather may be lower due to more extensive regions of low voltage and low baseline LI.

Combination of CF and LI monitoring may allow to improve ablation procedures in regions of low voltage: 1) LI mapping may complement electrogram-based substrate maps potentially facilitating selection of optimal ablation sites within previously ablated areas (Unger et al., 2021). This is supported by our findings confirming baseline LI as a better predictor for a ∆LI > 20 Ω compared to local voltage. 2) When ablating in areas of low voltage and encountering low baseline LI and ∆LI, combined CF and LI monitoring allows to discriminate between insufficient contact and low electrical coupling due to myocardial scar. A study analyzing conduction gaps acutely after index cavotricuspid isthmus ablation proposed that gaps could not be distinguished by baseline LI, whereas effective lesions showed higher ∆LI (Sasaki et al., 2020). Therefore, algorithms incorporating both CF and LI may enhance ablation efficacy (Schreieck et al., 2021), while combining CF and baseline LI may improve prediction of a sufficient ∆LI according to our analysis. Further studies are needed to investigate advanced ablation strategies incorporating individualized ∆LI and CF targets depending on anatomical region, baseline LI and local voltage.

### Limitations

The following limitations need to be addressed: First, this study reports an initial single-center experience with a relatively small sample size. Guidance of ablation using LI measurement was not performed prospectively. However, the amount of >700 analyzed RFC applications allowed to evaluate CF and LI dynamics. Second, as heterogenous regions of healthy and impaired myocardium were targeted during ablation, target ∆LI may vary from levels proposed in previous experimental and clinical studies investigating index procedures (Garrott et al., 2020; Szegedi et al., 2021; Ikenouchi et al., 2022; Sasaki et al., 2022) depending on local myocardial thickness, regional anatomical structures such as pouches and vessels as well as degree of fibrosis. Lastly, as follow-up data were not yet available, the impact of combined CF and LI assessment on long-term arrhythmia outcomes during repeat AF/AT ablation needs to be determined in future studies.

## Conclusion

Taken together, the present findings suggest that combining CF and LI may be useful to monitor real-time catheter-tissue electro-mechanical coupling and lesion formation within heterogenous atrial arrhythmia substrate in patients with repeat AF or AT ablation. Further studies are needed to assess the value for long-term outcome especially in challenging patient cohorts.

## Data Availability

The raw data supporting the conclusions of this article will be made available by the authors, without undue reservation.

## Abbreviations

AF, Atrial fibrillation; AT, Atrial tachycardia; AUC, Area under the curve; ∆Baseline LI, Difference of baseline and blood pool local impedance; CF, Contact force; CI, Confidence interval; LI, Local impedance; RFC, Radiofrequency current; ∆LI, Maximum local impedance drop during ablation; ∆LI%, Percentage of maximum local impedance drop compared to baseline local impedance.
